# Response of immunoglobulin M in gut mucosal immunity of common carp (*Cyprinus carpio*) infected with *Aeromonas hydrophila*

**DOI:** 10.3389/fimmu.2022.1037517

**Published:** 2022-11-17

**Authors:** Qingjiang Mu, Zhaoran Dong, Weiguang Kong, Xinyou Wang, Jiaqian Yu, Wei Ji, Jianguo Su, Zhen Xu

**Affiliations:** ^1^ Department of Aquatic Animal Medicine, College of Fisheries, Huazhong Agricultural University, Wuhan, Hubei, China; ^2^ State Key Laboratory of Freshwater Ecology and Biotechnology, Institute of Hydrobiology, Chinese Academy of Sciences, Wuhan, Hubei, China; ^3^ College of Fisheries and Life Science, Dalian Ocean University, Dalian, China; ^4^ Laboratory for Marine Biology and Biotechnology, Qingdao National Laboratory for Marine Science and Technology, Qingdao, China

**Keywords:** common carp, *Aeromonas hydrophila*, IgM, B cells, mucosal immune response

## Abstract

Immunoglobulin (Ig) M is an important immune effector that protects organisms from a wide variety of pathogens. However, little is known about the immune response of gut mucosal IgM during bacterial invasion. Here, we generated polyclonal antibodies against common carp IgM and developed a model of carp infection with *Aeromonas hydrophila via* intraperitoneal injection. Our findings indicated that both innate and adaptive immune responses were effectively elicited after *A. hydrophila* infection. Upon bacterial infection, IgM^+^ B cells were strongly induced in the gut and head kidney, and bacteria-specific IgM responses were detected in high levels both in the gut mucus and serum. Moreover, our results suggested that IgM responses may vary in different infection strategies. Overall, our findings revealed that the infected common carp exhibited high resistance to this representative enteropathogenic bacterium upon reinfection, suggesting that IgM plays a key role in the defense mechanisms of the gut against bacterial invasion. Significantly, the second injection of *A. hydrophila* induces strong local mucosal immunity in the gut, which is essential for protection against intestinal pathogens, providing reasonable insights for vaccine preparation.

## Introduction

Teleost fish are an important link in vertebrate evolution and an indispensable component of comparative immunology, which has both innate and adaptive immune systems (1). The adaptive immune system responds to a multitude of antigens and pathogenic microorganisms by producing specific immunoglobulins (Igs), which are more commonly known as antibodies. Igs secreted by B lymphocytes, an important immune effector, play a vital role in protecting fish against various pathogens (2). Igs come in two physical forms: the membrane-bound form located on the surface of B cells (i.e., B cell receptors [BCRs]) and the soluble form secreted by plasma cells (i.e., antibodies) (3). The main functions of BCRs, as antigen-bound receptors, are to bind, internalize, and target antigens and to present the antigenic peptide to helper T cells (4). The soluble form of Igs plays an important role in recognizing and binding to antigens to conduct various immune effector defensive processes, including antigen neutralization, complement activation, monocyte opsonization, and antibody-dependent cell-mediated cytotoxicity (ADCC) (5, 6).

Igs occur in vertebrates from cartilaginous fish to mammals (7). Five classes of Igs (i.e., IgM, IgD, IgG, IgA, and IgE) have been recognized in mammals. In contrast, only three Ig isotypes (i.e., IgM, IgD, and IgT) have been reported in teleost fish (8). IgM is the earliest identified Ig isotype, which exists predominantly as tetramers in serum and mucus (9, 10). Previous studies have shown that IgM concentrations are far higher compared to IgD or IgT in both systemic tissues and mucosal tissues of teleost fish (11, 12). Importantly, IgM has also known to involved in both systemic and mucosal immunity (13, 14). Furthermore, the membrane-bound IgM^+^ B lymphocytes of teleost fish are important subsets of B cells involved in innate and adaptive immune responses (15). Although IgD is the second Ig isotype discovered in teleost fish, its exact function is yet to be elucidated (16). A previous study demonstrated that IgD may enhance mucosal homeostasis (17). IgT, a recently identified member of the Ig family, is functionally equivalent to the IgA of mammals and is mainly involved in mucosal immunity (18). In addition, many antibodies against IgM have been developed in several teleost fish in recent years, which has greatly contributed to the development of fish Ig research (19–25). Although the IgM heavy chain gene of common carp (*Cyprinus carpio*) has been cloned (26), studies on IgM function are still lacking, especially in terms of the mucosal response to bacterial infection.

Common carp is among the major freshwater economic species worldwide, especially in China, with a total production of 2.8 million tons in 2021, exceeding one-tenth of the nation’s freshwater fish breeding production (27). Bacterial diseases such as *Aeromonas hydrophila*, *Aeromonas veronii*, and *Flavobacterium columnare* are important factors affecting the health and development of the current carp cultivation industry (28–30). *A. hydrophila* is a very common bacteria in aquatic environments worldwide (31). This opportunistic zoonotic bacterium can cause hemorrhagic septicemia, abdominal dropsy, and skin ulceration, and is considered the most devastating pathogen of *Cyprinoid* fish (28, 32). Previous studies have demonstrated that *A. hydrophila* induces intestinal inflammation in zebrafish and grass carp (33, 34). Moreover, the IgM expression in the intestine of rainbow trout was significantly increased after infection with *Streptococcus iniae* (35). However, the immune response and function of IgM against *A. hydrophila* infection in the common carp intestine remains largely unexplored.

To fill the aforementioned knowledge gaps, here we prepared polyclonal antibodies against the common carp IgM. Afterward, we developed a model of common carp infection with *A. hydrophila via* intraperitoneal injection. In this study, we found that *A. hydrophila* infection elicited a strong immune response and histopathologic changes in the gut. Notably, bacterial infection induced increases in IgM^+^ B cells and *A. hydrophila*-specific IgM titers in mucosal and systemic immunity. More importantly, our results demonstrated that common carp that were reinfected with *A. hydrophila* recovered faster from the pathological changes in their gut. Furthermore, *A. hydrophila*-specific IgM titers and survival rates were also higher in the reinfected carp. Therefore, the results in this paper provide crucial insights into the role of IgM in bacterial enteritis of teleost fish.

## Materials and methods

### Development of polyclonal antibody against common carp IgM

Completed IgM heavy chains of common carp cDNA sequences were obtained from NCBI (GenBank accession no. MH352354.1) and were synthesized artificially. We predicted the functional domains through the IMGT tool (http://www.imgt.org/3Dstructure-DB/cgi/DomainGapAlign.cgi). The heavy chain constant domains (CH2-CH4, 993bp) were amplified with primers F/R (F:5’-CGCGGATCCGATGTTCGCGCAACCGTT-3’ R:5’-CCGCTGGAGTTACGGTTTACAAAACGCCGG-3’) and 2×Taq Master Mix (Vazyme, China). The PCR products were purified using the FastPure^®^ Gel DNA Extraction Mini Kit (Vazyme, China), digested with restriction enzymes *Bam*H I and *Xho* I (Takara, Japan), and ligated to the pET-32a vector. Then the constructed plasmid was transformed into *Escherichia coli* BL21 (DE3) (Vazyme, China), inoculated on Luria-Bertani (LB) solid medium, and cultured at 37°C overnight. The expression and purification of recombinant IgM protein (rIgM) expression were performed by referring to the previous method of Wang et al. with minor modifications (36). Positive clones on the plates were then selected and inoculated in a 500 ml LB liquid medium (with 100 μg/ml ampicillin), then cultured at 37°C with shaking at 220 rpm. When the medium reached approximately 0.6 at OD600nm, isopropyl-β-d-thiogalactopyranoside (IPTG; Roche, Switzerland) was added at a final concentration of 1 mM and incubated at 28°C for 3.5 h. The rIgM was purified according to the manufacturer’s instructions by a nickel-nitrilotriacetic acid column (Ni-NTA; Qiagen, Germany). 400 μg purified rIgM and Freund’s Complete Adjuvant (FCA; Sigma, USA) were fully mixed in equal proportions and then used for injection immunization of Japanese white rabbits (2-3 months old). The rabbits were booster-immunized with 150 μg of purified rIgM mixed with Freund’s Incomplete Adjuvant (FIA; Sigma, USA) four times. After immunization, blood was collected from rabbits, and IgG was purified from rabbit serum with protein A agarose (Thermo Fisher Scientific, USA). Then we purified the obtained rabbit IgG by affinity chromatography to obtain the anti-carp IgM antibody. Briefly, the affinity column was prepared by coupling rIgM to CNBr-activated Sepharose 4B according to the manufacturer’s instructions (GE Healthcare, USA). For isolation anti-carp IgM pAb, the purified rabbit IgG sample was applied to the column equilibrated in 1× phosphate buffered saline (PBS, pH 7.2). Then incubate in the shaker for 2 h. After several washes of affinity column with PBS, bound IgM were eluted with 0.1 M glycine (pH 2.5), and immediately neutralized with 1 M Tris (pH 9.0). The neutralized antibody was displaced into PBS using a PD-10 Desalting Columns (GE Healthcare, USA) according to the manufacturer’s instructions and stored at -80°C. The specificity of the polyclonal antibody against common carp IgM was detected by western blot and immunofluorescence.

### Fish maintenance

The 10-15 g common carp used for this experiment were obtained from a fish farm in Chongqing, China, and placed in an aquarium containing a recirculating aquaculture system. Fish were fed with 153 commercial fish floating feed pellets (Tongwei Group, China) twice per day and acclimated at 28°C for at least 2 weeks. The feeding was terminated 48 h before injection and sampling. Animal procedures were approved by the Animal Experiment Committee of Institute of Hydrobiology, Chinese Academy of Sciences and carried out according to the relative guidelines.

### *A. hydrophila* strain and challenge test

*A. hydrophila* strain AH1 was obtained from the Laboratory of Aquatic Animal Medicine of the College of Fisheries at Huazhong Agricultural University (Hubei, China). The AH1 strain was streaked consecutively on LB solid medium and cultured at 28°C for 24 hours. Then, single colonies were picked and inoculated into LB liquid medium and cultured at 28°C for 12 h with 200rpm shaking. *A. hydrophila* suspension was centrifuged at 5000 g for 10 min, bacteria were resuspended with PBS and the concentration was adjusted to 1.6 × 10^7^ CFU/ml for challenge use. We designed two types of challenge experiments. The first was an infection of common carp by intraperitoneal injection with 100 μl of *A. hydrophila* (1.6 × 10^7^ CFU/ml), tissues (foregut, midgut, hindgut, spleen, and head kidney) from at least six fish were collected at 0.5, 1, 2, 4, 7, 14, 28 days post-infection (DPI), respectively, and fluids (serum and gut mucus) were taken on 28 DPI (**Figure 2A**). For the second type of challenge, a second intraperitoneal injection was performed with the same dose of *A. hydrophila* on day 28 after the first infection. Tissues, serum, and gut mucus were collected at 28 (28 DPI surviving group, [28DPI-S], before the second challenge) and 35 (35 DPI surviving group, [35DPI-S]) days post-primary infection. As the control group, the fish were injected with 100 μl of PBS. Tissue samples were used to evaluate morphological change and measure the expression of the immune-related genes, and serum and gut mucus were used to detect the changes in the IgM by western blot.

### Collection of serum and mucus

For common carp sampling, fish were anesthetized with MS-222. The blood was taken from the tail vein, centrifuged at 5000 g for 10 min to remove the plasma, collected the serum, and immediately stored at -80°C before use. To collect skin mucus, the mucus was gently scraped from the surface of common carp skin and then placed in a sterile Petri dish as previously described (37). To collect gut mucus, the gut of common carp was opened longitudinally, and the incubation buffer (1 ×PBS, containing 1 × protease inhibitor cocktail [Roche, Switzerland], 1mM phenylmethylsulfonyl fluoride [Sigma, Germany]) was added at a ratio of 4000 μl/g to the surface of the gut and then the surface mucus was gently scraped with a cell scraper to a sterile Petri dish. The sampling locations of foregut, midgut, and hindgut were shown in **Supplementary Figure 2B**. To obtain gill, buccal, nasal, and pharyngeal mucus, perfusion was performed using PBS to remove blood present in the tissue, and after dissection and removal of the tissue, it was rinsed three times with PBS to remove the remaining blood, added to the protease inhibition buffer described above (gill, added at a ratio of 2000 μl/g; buccal, added at a ratio of 10,000 μl/g; nasal, added at a ratio of 10,000 μl/g; pharyngeal, added at a ratio of 4000 μl/g), placed in sterile Petri dishes, and incubated overnight with gentle shaking at 4°C as previously described (38–41). After the tissue was gently blown, the culture supernatant was collected. All mucus suspensions were then collected into Eppendorf tubes and centrifuged at 400 g for 10 min at 4°C to remove existing the carp cells and debris. To remove bacteria from the mucus, the cell-free mucus was further centrifuged at 10,000 *g* for 10 min at 4°C and the mucus suspension was then stored at -80°C before use.

### SDS-PAGE and western blot

Mucus and serum samples were resolved on 4-15% SDS-PAGE Ready Gel (Bio-Rad, USA). PVDF membranes (Bio-Rad, USA) were first activated with methanol and the gels were transferred onto PVDF membranes for western blot analysis. PVDF membranes were blocked with 8% skim milk after the transfer and then incubated with anti-carp IgM (0.2 μg/ml) antibody. As isotype, the rabbit IgG (purified from rabbit blood before immunization) was used at the same concentrations. After washing four times, added HRP Goat Anti-Rabbit IgG (H+L) (ABclonal, China) and incubated for 45 min at RT. After washing four times, the ECL Substrate (Epizyme, China) was incubated with PVDF membranes. For quantitative analysis of IgM in serum and gut mucus, the PVDF membranes were scanned under the Amersham Imager 800 Imaging System (GE Healthcare, USA), and the signal intensity of each band was analyzed with ImageQuant TL software (GE Healthcare, USA). Thereafter, the concentration of IgM was determined by plotting the obtained signal strength values on a standard curve generated for each blot using known amounts of purified carp IgM. For purification of native IgM in common carp serum, the same procedure was used as for the purification of pAb as described above. Briefly, the affinity column was prepared by coupling anti-carp IgM pAb to CNBr-activated Sepharose 4B (GE Healthcare, USA). Then a serum sample pooled from several individual carp was diluted 1:2 in PBS (pH 7.2) was applied to the column equilibrated in PBS. The bound IgM was eluted in the same manner, and the concentration was determined using the Micro BCA™ Protein Assay Kit according to the manufacturer’s instructions (Thermo Fisher Scientific, USA).

### Histology, light microscopy, and immunofluorescence microscopy studies

As for histological studies, hematoxylin-eosin (H&E) staining was performed according to the previous method of Kong et al. (41). Images were captured for at least 6 individuals using a microscope, and then the length and width of at least 3 gut villi per image were measured using the measurement tool of cellSens Standard software (Olympus, Japan). IgM^+^ B cells were detected according to the method previously described by Yu et al. with minor modifications (40). Briefly, sections were subjected to antigen retrieval using EDTA-2Na, followed by incubation with rabbit anti-carp IgM pAb (1.5 μg/ml) overnight at 4°C. As isotype, the rabbit IgG (purified from rabbit blood before immunization) was used at the same concentrations. After washing four times with PBS, sections were stained with Goat anti-Rabbit IgG (H+L) Highly Cross-Adsorbed Secondary Antibody, Alexa Fluor™ Plus 488 (Invitrogen, USA) at 3.3 μg/ml each for 40 min at RT. After washing four times with PBS, all sections were stained with 1 μg/ml DAPI (4’, 6-diamidino-2-phenylindole; Invitrogen, USA) for 8 min at RT before mounting. All images were then captured using an Olympus BX53 fluorescence microscope (Olympus, Japan) and analyzed by cellSens Standard imaging processing software (Olympus, Japan).

### DNA or RNA isolation and quantitative real-time PCR analysis

DNA was extracted by using the DNeasy^®^ Blood & Tissue Kit (QIAGEN, German) according to the manufacturer’s protocol. Briefly, the tissues were cut up and mixed with 180 μl ATL and 20 μl proteinase K. The tissues were incubated at 56°C until they were completely lysed. Add 200 μl AL and incubate at 56°C for 10 min and then add 200 μl anhydrous ethanol. Transfer supernatant to DNeasy Mini spin column and centrifuge at 7000 g for 1 min. After washing the hybrids with AW1 and AW2, elution was performed with Buffer AE, collected the eluted DNA. The concentration of DNA was determined using a Nanodrop 2000 spectrophotometer (Thermo Fisher Scientific, USA). To test bacterial loads, we selected Act virulence genes from *A. hydrophila* for qPCR. Total RNA was extracted by homogenization in 1 ml TRIzol (Invitrogen, USA) using steel beads and shaking (60 HZ for 1 min; WONBIO, China) following the manufacturer’s instructions. Extracted RNA was measured by spectrophotometer for concentration and checked for integrity with agarose gel (Monad, China). To eliminate differences in gene expression levels for each sample, RNA was reverse transcribed to cDNA and then analyzed by qPCR according to the method of Yu et al. (40). The relative mRNA levels of IgM in different tissues were calculated by taking the reciprocal of the value of IgM/40S. The relative fold changes were calculated using the 2^-ΔΔCt^ method, and 40S was used as a control gene for normalization of expression. The specific primers used in this experiment are listed in **Supplementary Table 1**.

### Binding of common carp IgM to *A. hydrophila*

Using the pull-down assay as described previously (42, 43), we measured the titers of *A. hydrophila*-specific IgM in foregut mucus, midgut mucus, hindgut mucus, and serum. Briefly, the suspension of *A. hydrophila* cultured to OD600nm = 0.7 was washed three times with PBS. Then 40 μl of bacterial suspension were added to the incubation system, and the mucus was diluted at 1:2, 1:10, 1:40, 1:100, and the serum was diluted at 1:10, 1:100, 1:1000, 1:4000, respectively, and the system was supplemented to 300 μl with 1% BSA-PBS. After incubation at RT for 4 h or at 4°C for 12h, the supernatant was discarded by centrifugation at 10,000 *g* for 5 min and the sediment was washed three times with PBS. Add 2 × Laemmli Sample Buffer (Bio-Rad, USA) to elute bound proteins and mix well, boil at 95°C for 8 min. The prepared samples were separated by SDS-PAGE and then detected for the presence of IgM using western blot and the polyclonal antibody against common carp IgM as described above.

### Statistical analysis

The data were shown as the mean ± SEM and normality and homogeneity of variance were checked before the statistical analysis. The statistical differences between groups were analyzed using an unpaired Student’s *t*-test, one-way analysis of variance (ANOVA) with Bonferroni correction, and log-rank (Mantel-Cox) test (Prism version 6.01; GraphPad). A value of *p* < 0.05 or less was considered statistically significant.

## Results

### Antibody preparation and validation of antigen recognition

**Figure 1:**
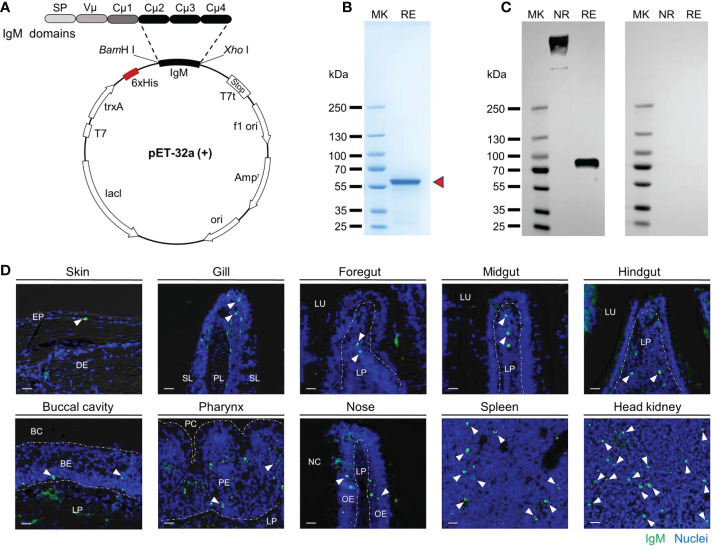
The common carp IgM polyclonal antibody (pAb) production and characterization. **(A)** Structural schematic of pET-32a-IgM recombinant plasmids. **(B)** Coomassie blue staining of recombinant protein of IgM resolved by SDS-PAGE. Red arrowheads indicate the recombinant IgM. **(C)** Western blot analysis with pAb to common carp serum. Left margin, molecular size in kilodaltons (kDa). MK, marker; NR, nonreducing conditions; RE, reducing conditions. **(D)** Immunofluorescence staining of IgM^+^ B cells in different tissues of common carp. Tissue paraffinic sections were stained with rabbit anti-carp IgM (green) and nuclei were stained with DAPI (blue) (isotype-matched control antibody staining, **Figure S1A**). White arrowheads point to cells stained for IgM. White dotted lines outline the border. Scale bar, 20 μm. EP, epidermis; DE, dermis; PL, primary lamella; SL, secondary lamella; LU, lumen; LP, lamina propria; BC, buccal cavity; BE, buccal epithelium; PC, pharyngeal cavity; PE, pharyngeal epithelium; NC, nasal cavity; OE, olfactory epithelium.

To prepare polyclonal antibodies against common carp IgM, we first obtained the complete cDNA sequence of IgM heavy chain from NCBI (GenBank accession: no. MH352354.1) and cloned the CH2-CH4 domains into the prokaryotic expression plasmid (**Figure 1A**). Then we purified and identified the soluble expression products. As we expected, the purified recombinant proteins exhibited the main band with molecular weights of ~56 kilodaltons (kDa) (**Figure 1B**). The rIgM was then used as an immunogen to develop against common carp IgM antibody. The rabbit polyclonal antibody (pAb) against common carp IgM was analyzed by western blot using common carp serum dilution. The results showed significant bands at ~800 kDa and ~75 kDa under non-reducing and reducing conditions, respectively, and no bands for the antibody (rabbit IgG) of the isotype control (**Figure 1C**). By immunofluorescence assay, we found that green fluorescent signals were observed on histological sections from the skin, gill, foregut, midgut, hindgut, buccal cavity, pharynx, nose, spleen, and head kidney of the healthy common carp, and isotype control was negative, which suggested that the pAb could specifically react with membrane-bound IgM-positive B lymphocytes (**Figure 1D** **;** **Supplementary Figure 1A**).

We also investigated the expression pattern of *igm* in healthy common carp by qPCR. As shown that *igm* expression was found in all examined tissues and measured at higher levels in the systemic tissues (i.e., head kidney and spleen) than in the mucosal tissues (i.e., skin, gill, foregut, midgut, hindgut, buccal cavity, pharynx, and nose) (**Supplementary Figure 1B**). In addition, the concentrations of IgM in serum and mucus were examined at the protein level in this study, and we found that the concentration of IgM in mucus was much lower than in serum (**Supplementary Figure 1C**). Together, these data indicated that the carp IgM pAb herein was workable and could be used for western blot and immunofluorescence analysis.

### *A. hydrophila* infection induces severe pathological changes in common carp

**Figure 2:**
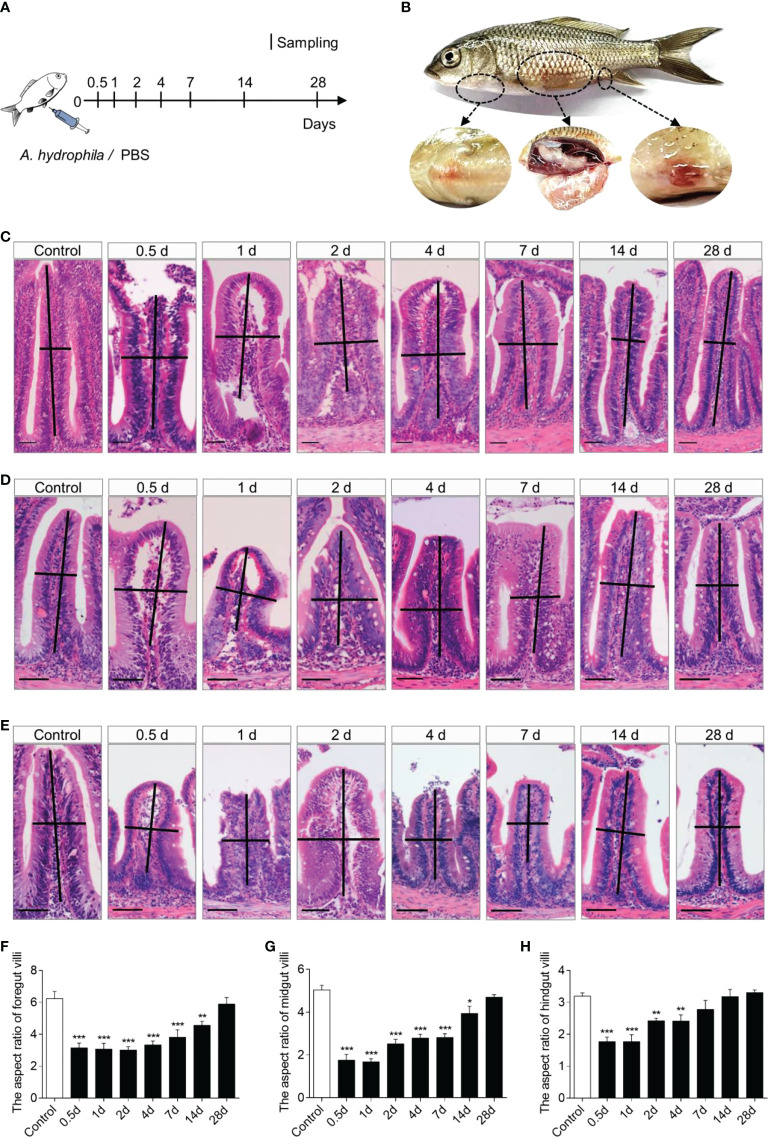
Scheme of the infection strategy with *A*. *hydrophila via* intraperitoneal injection. **(A)** Fish were injected with *A. hydrophila* and sacrificed at 0.5, 1, 2, 4, 7, 14, and 28 DPI for sample collection, while fish were injected with PBS as control. **(B)** The clinical observation following challenge with *A. hydrophila*. **(C–E)** Histological examination by H&E staining of foregut **(C)**, midgut **(D)**, and hindgut **(E)** from control and infected fish (*n* = 6). Scale bars, 50 μm. **(F–H)** The length-width ratio of foregut **(F)**, midgut **(G)**, and hindgut **(H)** intestinal folds in these groups of fish from **(C–E)** (*n* = 6). *P < 0.05, **P < 0.01, and ***P < 0.001 (one-way ANOVA with Bonferroni correction). Data are representative of at least three independent experiments (mean ± SEM).

To evaluate the common carp immune responses to bacterial pathogenic challenges, we developed a common carp bacterial infection model *via* intraperitoneal injection of *A. hydrophila* (**Figure 2A**). In the infected group, typical symptoms of *A. hydrophila* appeared, such as ascites, anal redness and swelling, body congesting, and enteritis (**Figure 2B**). In addition, we found that the walls of the foregut, midgut, and hindgut were congested (**Supplementary Figure 2A**). Using H&E staining, we observed severe pathological changes in the gut of common carp after *A. hydrophila* infection, including gut villus damage as well as a decrease in the gut villus aspect ratio (**Figures 2C–E**). The aspect ratio of gut villi reduced significantly at 0.5 DPI and gradually recovered to the normal level until 28 d (**Figure 2F, G** **)**. Notably, the hindgut recovery was faster than foregut and midgut, with no significant difference in the infected group compared with control fish at 7 DPI (**Figure 2H**). Together, our results confirmed that the *A. hydrophila* infection model was successful in the common carp.

### Response of immune-related genes in common carp after infection with *A. hydrophila*

**Figure 3:**
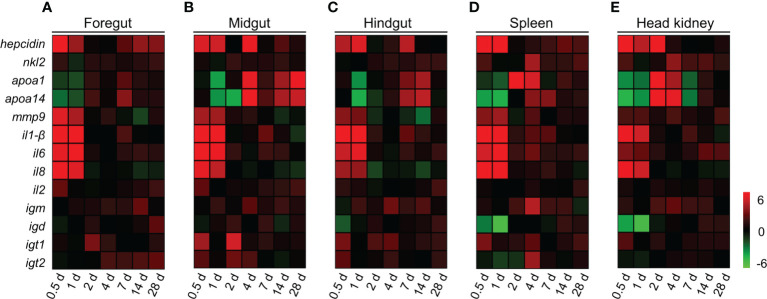
Immune response in gut, spleen, and head kidney tissues of common carp following *A. hydrophila* infection. **(A–E)** Heat map illustrates results from qPCR of mRNAs for selected immune-related genes in bacteria-challenged fish vs. control group measured at 0.5, 1, 2, 4, 7, 14, and 28 DPI following with *A. hydrophila* in the foregut **(A)**, midgut **(B)**, hindgut **(C)**, spleen **(D)**, and head kidney **(E)** organs of common carp (*n* = 6). Color value: log_2_ (fold change). Data are representative of at least three different independent experiments.

To evaluate the kinetics of immune responses in common carp post *A. hydrophila* infection, we measured the expression levels of 13 immune-related genes in the foregut, midgut, hindgut, spleen, and head kidney tissues of common carp at 0.5, 1, 2, 4, 7, 14, and 28 DPI by qPCR, which including antimicrobial peptides (AMPs) genes (*hepcidin*; NK-lysin 2, *nkl2*; Apolipoproteins A-1, *apoa-1*; and Apolipoproteins A-14, *apoa-14*), inflammatory genes (inflammatory 1-β, *il1-β*; inflammatory 6, *il6*; inflammatory 8, *il8*; and inflammatory 2, *il2*), matrix metalloproteinase 9 (*mmp-9*), and immunoglobulin (Ig) heavy chain genes (*igm*, *igd*, *igt1*, and *igt2*). In this study, we found that most of the genes increased significantly in foregut, midgut, hindgut, spleen, and head kidney tissues after *A. hydrophila* infection (**Figures 3A–E**). As AMPs, the expression of *hepcidin* and *nkl2* were upregulated significantly at 0.5 DPI. Interestingly, the *apoa-1* and *apoa-14* genes decreased significantly at first (0.5 and 1 DPI) and then increased significantly. The higher expression levels of proinflammatory cytokines, such as *il1-β*, *il6*, and *il8*, were also expressed at 0.5 and 1 DPI. However, the inflammatory cytokine *il2* was significantly upregulated at the initial phase of inflammation in common carp gut, as well as at the later phase of tissue remodeling (**Figures 3A–C**). It is worth noting that the genes of *igm* and *igt1* were expressed earlier than *igd* and *igt2* in the spleen and head kidney (**Figures 3D, E** **)**, and the up-regulation of *igm* expression can persist up to 14 days after *A. hydrophila* infection. In addition, the expression of *igm* and *igt2* were upregulated in the foregut, midgut, and hindgut post the second infection (**Supplementary Figure 3**). Taken together, these data supported a strong innate and adaptive immune response were generated in systemic and mucosal tissues following intraperitoneal infection with *A. hydrophila* in common carp.

### Response of IgM^+^ B cells to *A. hydrophila* infection

**Figure 4:**
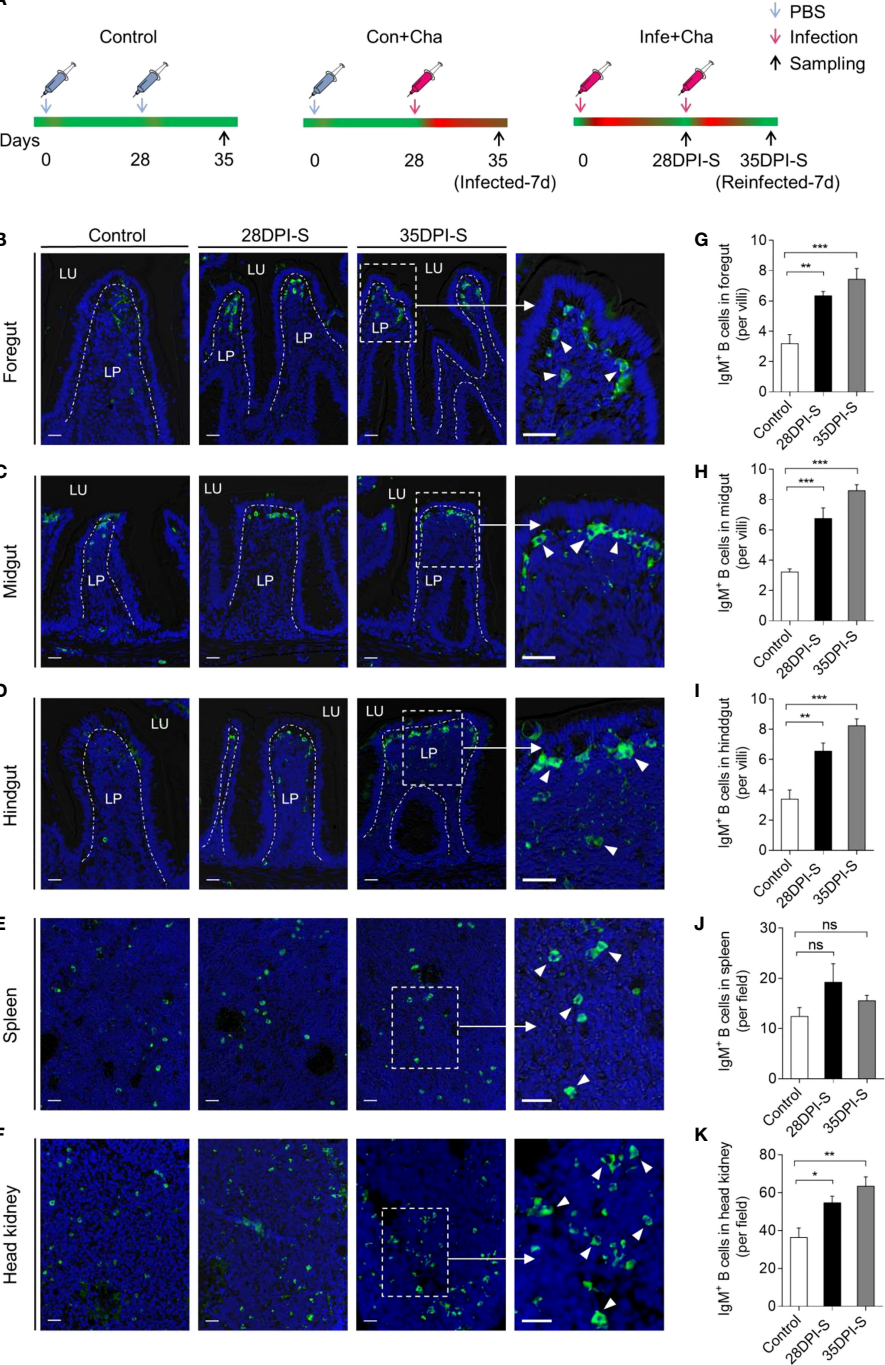
Scheme of the reinfection strategy and accumulation of IgM^+^ B cells of common carp infected with *A. hydrophila*. **(A)** Scheme of the rechallenge strategy with *A. hydrophila via* intraperitoneal injection. Fish were injected with *A. hydrophila* at 0 days and reinfected at 28 DPI with the same bacterial dose, and the resulting surviving fish were sacrificed at 28 and 35 days after the primary infection (28 DPI surviving group, [28DPI-S]) and 35 (35 DPI surviving group, [35DPI-S]). The three groups of fish include Control (PBS + PBS), Con+Cha (PBS + *A. hydrophila*), and Infe+Cha (*A. hydrophila* + *A*. *hydrophila*). **(B–F)** Representative differential interference contrast (DIC) images of immunofluorescence staining on common carp foregut **(B)**, midgut **(C)**, hindgut **(D)**, spleen **(E)**, and head kidney **(F)** paraffin-sections from uninfected (Control) fish, 28DPI-S, and 35DPI-S fish, stained for IgM (green) (Isotype-matched control antibody staining is shown in **Figure S1A**); nuclei (blue) were stained with DAPI (blue). Scale bars, 20 μm. **(G–K)** The number of IgM^+^ B cells of Control, 28DPI-S, and 35DPI-S fish counted from **(B–F)** (*n* = 6). ns, not significant, *P < 0.05, **P < 0.01, and ***P < 0.001 (unpaired Student’s *t*-test). Data are representative of at least three independent experiments (mean ± SEM).

To evaluate the adaptive IgM^+^ B cells responses, we performed a second intraperitoneal challenge of common carp using the same dose of *A. hydrophila* as the first at 28 DPI (**Figure 4A**). Using immunofluorescence microscopy, the results showed a significant increase in the number of IgM^+^ B cells in the foregut, midgut, and hindgut of 28DPI-S fish, which were ∼2.1-, ∼2.1-, and ∼2.0-fold higher compared to the control fish (**Figures 4B–D, G–I**). And we detected a moderate increase of IgM^+^ B cells in the head kidney of the 28DPI-S fish, which was ∼1.5-fold higher when compared to control group (**Figure 4F, K**). Notably, 35DPI-S fish showed significantly increased numbers of IgM^+^ B cells in the foregut, midgut, hindgut, and head kidney, which were ∼2.5-, ∼2.7-, ∼2.5-, and ∼1.8-fold, respectively, when compared to the control fish (**Figures 4G–I, K** **)**. However, the trend of IgM^+^ B cells in the spleen was not significant compared to the control fish (**Figure 4E, J**).

### Bacteria-specific IgM responses in gut mucus and serum

**Figure 5:**
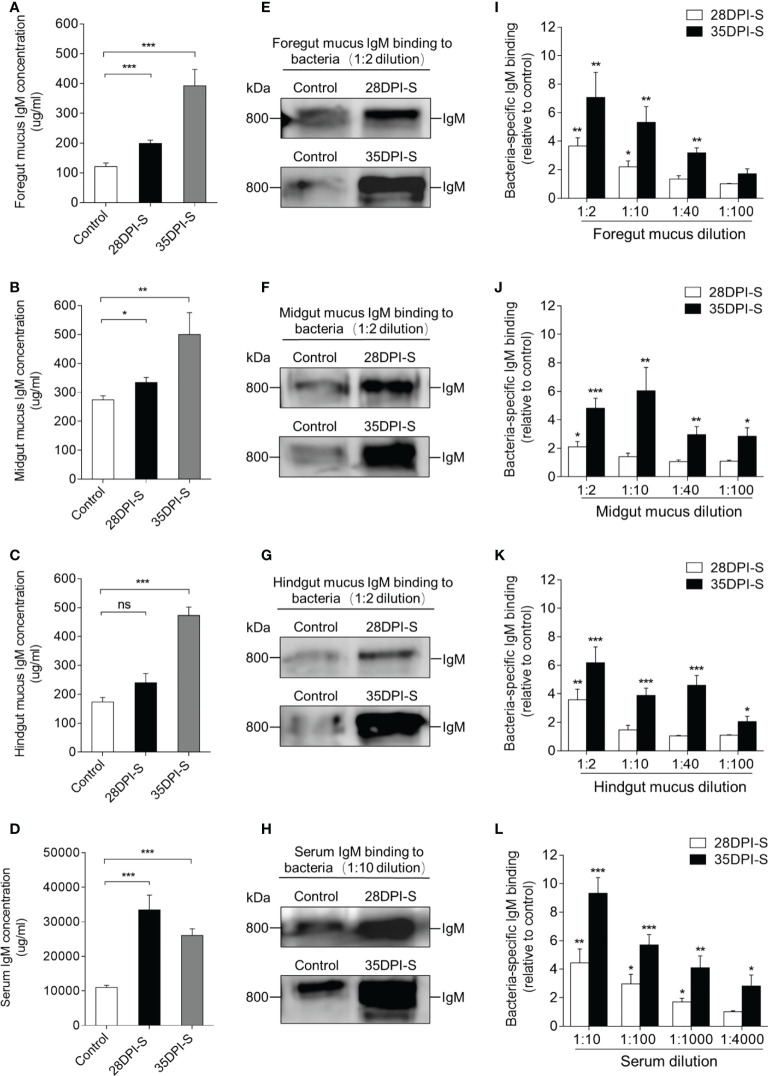
IgM responses in the gut mucus and serum from 28DPI-S and 35DPI-S fish. **(A–D)** The concentration of IgM protein in foregut mucus **(A)**, midgut mucus **(B)**, hindgut mucus **(C)**, and serum **(D)** of Control, 28DPI-S, and 35DPI-S fish (*n* = 6). **(E–H)** Western blot analysis of IgM-specific binding to *A*. *hydrophila* in foregut mucus **(E)**, midgut mucus **(F)**, hindgut mucus **(G)**, and serum **(H)** (mucus dilution 1:2; serum dilution 1:10) from 28DPI-S and 35DPI-S fish. **(I–L)** IgM-specific binding to *A. hydrophila* in dilutions of foregut mucus **(I)**, midgut mucus **(J)**, hindgut mucus **(K)**, and serum **(L)** from 28DPI-S and 35DPI-S fish, evaluated by densitometric analysis of immunoblots and presented as relative values to those of control fish (*n* = 6). ns, not significant, *P < 0.05, **P < 0.01, and ***P < 0.001 (unpaired Student’s *t*-test). Data are representative of at least three independent experiments (mean ± SEM).

To further study the gut bacteria-specific IgM responses in common carp after *A. hydrophila* infection, we performed a western blot analysis. Here, the results showed that the concentration of IgM in the foregut and midgut mucus of the 28DPI-S fish increased moderately and significantly (**Figures 5A, B** **)**. The protein level of IgM in the foregut mucus, midgut mucus, and hindgut mucus of 35DPI-S fish was increased significantly, which was ∼3.2-, ∼1.9-, and ∼2.8- fold higher than that of the control fish, respectively (**Figures 5A–C**). In serum, the concentrations of IgM increased ∼3.0-fold and ∼2.4-fold in 28DPI-S and 35DPI-S fish, respectively (**Figure 5D**). The increase in the level of IgM protein led us to hypothesize that IgM plays an important role in the defense against bacterial invasion of the organism. Next, we verified the above hypothesis using a pull-down assay to detect the ability of IgM to bind to *A. hydrophilic* in serum and gut mucus. Immunoblot analysis showed that bacteria-specific IgM binding in a 1:2 midgut mucus (∼2.1-fold) and hindgut mucus (∼3.5-fold) dilution from 28DPI-S fish and up to 1:100 in dilutions of midgut mucus and hindgut mucus in 35DPI-S fish, an increase of ∼2.8-fold and ∼2.1-fold, respectively, compared to control fish (**Figures 5F, G, J, K** **)**. We found that bacteria-specific IgM binding was detected at 1:10 (∼2.2-fold) foregut mucus dilution in the 28DPI-S fish, in contrast to midgut and hindgut results where the binding was detected only at 1:40 (∼3.1-fold) foregut mucus dilution in the 35DPI-S fish (**Figures 5E, I** **)**. In addition, there was a significant increase in binding of bacteria-specific IgM in the diluted serum of the 28DPI-S fish and 35DPI-S fish compared with control fish, reaching 1:1000 (∼1.8-fold) and 1:4000 (∼2.8-fold), respectively (**Figures 5H, L** **)**. In a word, these results indicated that IgM plays an important role in the gut tissue against bacterial invasion.

### Reinfected fish exhibit resistance to *A. hydrophila*

**Figure 6:**
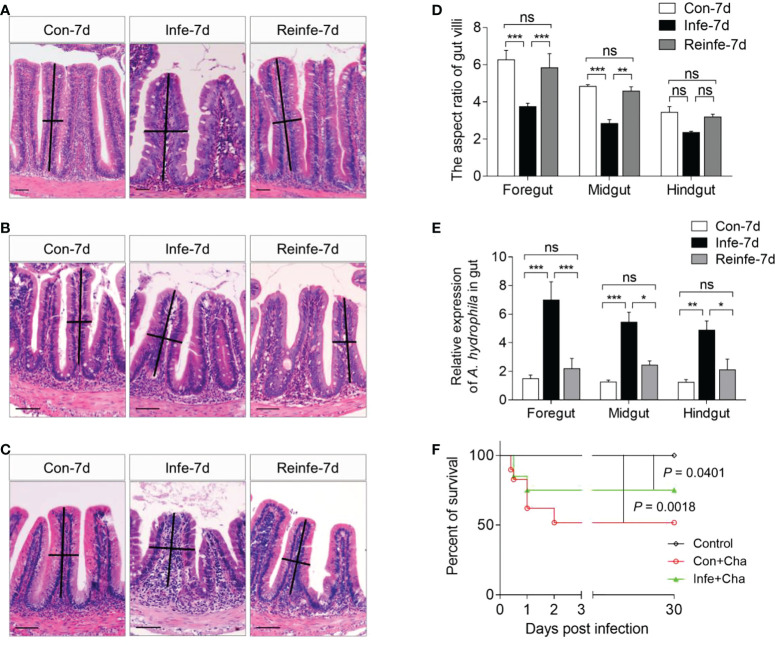
Histological examination, bacterial loads, and survival rates of common carp after rechallenging with *A. hydrophila*. **(A–C)** Histological examination by H&E staining of foregut **(A)**, midgut **(B)**, and hindgut **(C)** from Control (Con-7d), Con+Cha (Infe-7d), and Infe+Cha (Reinfe-7d) groups of fish. **(D)** The length-width ratio of foregut, midgut, and hindgut intestinal folds from **(A–C)**. **(E)** The expression levels of *A. hydrophila* in control, infected-7d, and reinfected-7d fish were measured in the gut of common carp (*n* = 6). **(F)** Cumulative survival of control, infected, and reinfected fish. Statistical differences were evaluated by log-rank (Mantel-Cox) test. ns, not significant, *P < 0.05, **P < 0.01, and ***P < 0.001 (one-way ANOVA with Bonferroni correction). Data are representative of at least three independent experiments (mean ± SEM).

In reinfection model, common carp were euthanized at 35 DPI to evaluate pathological changes in the gut tissues and bacterial loads in the foregut, midgut, hindgut, spleen, and head kidney tissues from the three groups (Control, Con+Cha, Infe+Cha) (**Figures 6A–E**). Using H&E staining, we found that the aspect ratio of gut villi was not significantly different between control fish and reinfected-7d fish (**Figure 6D**). By qPCR, we detected that upon reinfection, the reinfected fish had markedly lower cytotoxic enterotoxin (Act) expression of *A. hydrophila* compared with those of the first infected fish (**Figure 6E**, **Supplementary Figure 4**). Importantly, we found a higher survival rate in the Infe+Cha group compared with the Con+Cha group (**Figure 6F**). These data strongly suggested that pre-infection with *A. hydrophila* protects common carp from the bacterial reinfection, and mucosal adaptive immunity had been induced in common carp against bacteria invasion.

## Discussion

Fish live in complex aqueous environments and are therefore uniquely susceptible to diseases caused by a variety of pathogens (44). In response to these threats, teleost fish have evolved robust innate and adaptive immune systems, thus ensuring an appropriate immune response upon antigen challenge while maintaining overall tissue and microbiota homeostasis (45–47). Igs are important effector molecules of the adaptive immune system in vertebrates, which play critical roles in defending the host against various pathogens (48). In teleost fish, IgM is the most abundant Ig isotype and plays a crucial role in both systemic and mucosal tissues (13, 14). The gut of teleost fish represents the prevalent mucosa-associated lymphoid tissue (MALT), directly interacts with foreign pathogens from aquatic environment, and is often considered the major site of invasion (49). *A. hydrophila* is a highly infectious enteropathogenic bacterium commonly found in freshwater, which causes intestinal inflammation and hemorrhagic septicemia, resulting in considerable economic losses (50). However, the IgM function in the development of bacterial enteritis in teleost fish is relatively little known. Here, we generated polyclonal antibodies against common carp IgM to investigate the function of gut IgM in protecting against enteropathogenic bacterial invasion.

Antibodies against IgM have been reported in much teleost fish (19–25). In this study, the rIgM was employed as an immunogen to generate polyclonal antibodies. Under reducing conditions, the results showed that the molecular weight of rIgM was approximately 56 kDa, indicating that we successfully expressed the rIgM protein in *E. coli*. Generally, IgM occurs as tetramers with a molecular weight of approximately 800 kDa in the teleost fish, and its monomer consists of two heavy chains (~75 kDa) and two light chains (~25 kDa) (19, 24, 51–53). In our study, western blot analysis revealed that the polyclonal antibody (pAb) against carp IgM specifically reacted with the band corresponding to the heavy chain (~75 kDa) as expected, and IgM predominantly occurred as tetramers (~800 kDa) in common carp serum, which is consistent with the previous study (54). Moreover, the immunofluorescence results indicated that the IgM pAb could specifically recognize the IgM^+^ B cells of common carp. Notably, the qPCR and immunoblotting results showed that the expression and concentration of IgM in mucosal tissues were lower than those in systemic tissues, which was consistent with previous results (25, 37, 38, 40, 55). Overall, these findings indicated that the IgM pAb prepared herein could be used as a monitoring tool to assess the immune status of common carp.

To evaluate the immune responses of IgM in the gut against bacterial invasion, we established an *A. hydrophila* infection model in common carp *via* intraperitoneal injection. As expected, the fish exhibited the typical symptoms of *A. hydrophila* infection, including body surface hemorrhage, abdominal dropsy, and enteritis (28). Moreover, severe histological changes occurred in the gut of common carp upon infection, including gut villi shedding and lower aspect ratio of the villus structure, which was consistent with the known symptoms of *A. hydrophila*-infected grass carp (32). Notably, we found that the most serious symptoms occurred at 0.5-1 DPI, after which they gradually improved, indicating that *A. hydrophila* is an acute infectious gastroenteritis type bacterium. Overall, our results demonstrated that *A. hydrophila* successfully induces intestinal inflammation *via* intraperitoneal injection.

Innate immunity serves as the first line of defense against pathogen invasion. Upon *A. hydrophila* infection, consistent with the gut significant histopathological lesions, the results showed that six immune-related genes were significantly up-regulated in the foregut, midgut, hindgut, spleen, and head kidney, as early as 0.5 DPI. Hepcidin, an important antimicrobial peptide (AMP), plays a crucial role in host defense against bacterial invasion (56). Here, we found that the mRNA expression levels of *hepcidin* were quickly up-regulated in the foregut, midgut, hindgut, spleen, and head kidney post *A. hydrophila* infection. This demonstrated that hepcidin may be an important component of the innate immunity of carp and participates in mucosal and systemic immune responses against bacterial infection. NK-lysin is a potent antimicrobial peptide that is widely distributed in vertebrates (57). Upon *A. hydrophila* infection, the *nkl2* gene was significantly activated, suggesting that Nkl2 plays an important role in the immune response against microbial pathogens of common carp. Matrix metalloproteinase 9 (Mmp-9), a member of the zinc-dependent endopeptidase family, is associated with vital inflammatory processes in mammals (58). At the beginning of infection, significant up-regulation of *mmp*-9 expression was detected in the foregut, midgut, hindgut, spleen, and head kidney, suggesting the important role of Mmp-9 in the innate immune response. Moreover, the mRNA expression of proinflammatory cytokines such as *il1-β*, *il6*, and *il8* was also significantly increased in the foregut, midgut, and hindgut of common carp immediately after *A. hydrophila* infection. The results were similarly reported in zebrafish infected with *Staphylococcus aureus* and in common carp with SMB-induced enteritis (59, 60). Apolipoprotein A (Apo A) is a multifunctional protein that participates in lipid metabolism and transport. A previous study demonstrated that Apo A-1 has bacteriostatic activity against *A. hydrophila* and bactericidal activity against *E. coli* (61). Interestingly, *apoa-1* and *apoa-14* were first downregulated and then upregulated during the infection process, which was consistent with the findings of a previous study in carp infected with SVCV (62). The expression of *apoa-1* and *apoa-14* were upregulated in the foregut, midgut, and hindgut of common carp post the second infection. Overall, our results suggested that Apo A plays an important role in the resistance to invasion by *A. hydrophila*.

Similar to mammals, adaptive immunity in teleost fish plays a vital role in preventing bacterial infection (42, 63). Previous studies have shown that IL2 has regulatory effects on immune gene expression and may be involved in the differentiation of Treg and Th1/2 cells, in addition to enhancing NK cell activity (64, 65). Interestingly, *il2* upregulation occurred both at the early and late stages in the gut, indicating that IL2 plays an indispensable role in both innate and adaptive immunity in fish mucosal tissues. Igs are crucial components of adaptive immunity, and the expression of *igm*, *igd*, and *igt* was up-regulated in the foregut, midgut, hindgut, spleen, and head kidney tissues in this study. Interestingly, although the *igm* responses in the spleen and head kidney were activated earlier than in gut tissues, up-regulation of *igm* expression was also observed in the gut during the later phase of tissue remodeling. Meanwhile, upregulation of *igm* expression occurred in the foregut, midgut, hindgut, spleen, and head kidney post the second infection, which suggested that the IgM plays a vital role in adaptive immunity. Taken together, our results showed that both innate and adaptive immune responses could be effectively elicited after *A. hydrophila* infection.

A previous study showed that the expression of *igm* increased dramatically in gut of rainbow trout to which *Flavobacterium psychrophilum* was administered *via* intraperitoneal injection, and IgM was a main participant in the adaptive immune response to this pathogen challenge (66). Similar to mammals, the adaptive immune system of teleost fish also possesses immune memory, which produces a rapid and strong immune response upon pathogen reinfection (67). Thus, we developed a reinfection model, which demonstrated the induction of adaptive immune responses in the gut. In brief, IgM^+^ B cells were significantly increased in the foregut, midgut, hindgut, and head kidney of the 28DPI-S fish and 35DPI-S fish compared to the control fish. Notably, the accumulation of IgM^+^ B cells was also increased in the spleen, albeit not significantly, which may be due to the migration of cells into the gut from the spleen (68, 69). A large number of IgM^+^ B cells were detected in the gut of 35DPI-S fish, which coincided with increases in IgM concentration in the gut mucus of the same individuals. Higher bacteria-specific IgM titers were found in the gut mucus and serum in the 35DPI-S fish when compared to the 28DPI-S fish, indicating that IgM plays an important role in both systemic and mucosal immunity. Similarly, a study performed by Castro et al. showed that IgM^+^ B cells increased significantly in the peritoneal cavity of rainbow trout after injection with *E. coli* and viral hemorrhagic septicemia virus (VHSV), and these cells increased their IgM secretion (70). In contrast, bacteria-specific IgM titers were not found in the mucosal tissues (i.e., skin, gill, and buccal cavity) of rainbow trout after *F. columnare* infection by immersion in our previous studies (42, 43, 71). In another study that examined the GALTs of rainbow trout that had survived *Ceratomyxa shasta* infection *via* immersion, parasite-specific IgM responses were only detected in serum samples (18). Based on these observations, we hypothesized that IgM responses vary in an infection strategy-specific manner. Furthermore, we found that the expression of *igt* was stronger in the gut than in the spleen and head kidney after bacterial infection, especially after the second infection, implying that IgT plays an important role in the mucosa to defend against *A. hydrophila* infection. This finding is in agreement with the results of Zhang et al. and also applies to the skin, gill, and oral mucosal tissues, and in later work, we will develop antibodies against common carp IgT for further validation (18, 42, 43, 71).

More importantly, our previous studies demonstrated that the survivor rainbow trout re-exposed to this bacterium or virus exhibited high resistance (42, 72). In this study, we found that common carp reinfected with *A. hydrophila* exhibited higher survival rates and the pathological changes in the gut recovered faster compared to those in individuals infected for the first time. These findings suggested that local humoral immunity might be elicited in carp GALT thus contributing to protection upon reinfection. After rechallenging with *A. hydrophila*, based on the significant decrease in bacterial load, we hypothesized that IgM^+^ B cells secrete *A. hydrophila*-specific IgM to neutralize the bacteria, thereby preventing the pathogens from replicating and multiplying in the target cells. However, further studies are required to explain the mechanisms through which IgM neutralizes pathogens. Moreover, previous studies have demonstrated that *A. hydrophila* has developed resistance to commercial antibiotics, thus highlighting the importance of vaccines as an effective prevention measure (50). Injection is the most common route of vaccine administration, and therefore it is important to determine whether local mucosal immunity in the gut can be induced by injection. Previous studies hypothesized that the immune responses induced by mucosal (gut) and parenteral immunization are asymmetrical (73). In a previous study, when antigens were delivered *via* the gut, mucosal and systemic immune responses were elicited, as demonstrated by an increase in the amounts of circulating IgM (74). In contrast, delivering the dinitrophenol (a model antigen) by intraperitoneal injection resulted in strong systemic responses, whereas mucosal responses were virtually absent (75). However, our results demonstrated that intraperitoneal injection with *A. hydrophila* elicited a strong mucosal and systemic immune response in common carp. In other words, extraintestinal exposure to enteropathogenic bacteria triggered both immune responses equally in our infection model. Therefore, the above-described results suggested that IgM^+^ B cell and IgM responses may vary in a pathogen-specific manner. From a practical perspective, further research on the pathogen-specific responses of IgM in fish mucosa would greatly contribute to improving delivery strategies and adjuvant formulations. In turn, this would enable the creation of more effective vaccines to induce the generation of specific antibodies in the plasma cells of fish.

In conclusion, our study successfully developed a pAb against common carp IgM, which can be used as an effective tool for vaccine evaluation. Moreover, we constructed a model of carp infected with *A. hydrophila via* intraperitoneal injection, which can cause strong systemic and mucosal immunity. Importantly, our results demonstrated that reinfected common carp had a lower bacterial load, higher survival rates and higher bacteria-specific IgM titers compared to the common carp that had only been infected once. Therefore, IgM memory B cells may resist the invasion of *A. hydrophila* by being rapidly and massively activated and exerting various functions. Furthermore, our results suggest that IgM immune responses could vary in different infection strategies. Nevertheless, additional studies are required to elucidate the mechanisms through which IgM participates in the response against bacterial invasion in common carp.

## Data availability statement

The original contributions presented in the study are included in the article/**Supplementary Material**. Further inquiries can be directed to the corresponding author.

## Ethics statement

The animal study was reviewed and approved by the Animal Experiment Committee of Institute of Hydrobiology, Chinese Academy of Sciences.

## Author contributions

QM and ZD performed most of the experiments and wrote the manuscript. QM analyzed the data. WK designed infection model and revised manuscript. XW and JY helped with most of the experiments. WJ, JS, and ZX designed the experiments and revised the manuscript. All authors contributed to the article and approved the submitted version.

## Funding

This work was supported by grants from the National Natural Science Foundation of China (32225050, 32073001, and 31873045) and a grant from the State Key Laboratory of Freshwater Ecology and Biotechnology (2022FB13).

## Conflict of interest

The authors declare that the research was conducted in the absence of any commercial or financial relationships that could be construed as a potential conflict of interest.

## Publisher’s note

All claims expressed in this article are solely those of the authors and do not necessarily represent those of their affiliated organizations, or those of the publisher, the editors and the reviewers. Any product that may be evaluated in this article, or claim that may be made by its manufacturer, is not guaranteed or endorsed by the publisher.
