# Supplementary material for: Response of immunoglobulin M in gut mucosal immunity of common carp (Cyprinus carpio) infected with Aeromonas hydrophila
Source: Front Immunol. 2022 Nov 17;13:1037517. doi: 10.3389/fimmu.2022.1037517 (PMC9713697; doi:10.3389/fimmu.2022.1037517)
Supplement: Supplementary file 1 [file DataSheet_1.docx]

**Supplementary Information**

**Response of immunoglobulin M in gut mucosal immunity of common carp (*Cyprinus carpio*) infected with *Aeromonas hydrophila***

Qingjiang Mu^1†^, Zhaoran Dong^1†^, Weiguang Kong^2^, Xinyou Wang^1,2^, Jiaqian Yu^2,3^, Wei Ji^1^, Jianguo Su^1^ and Zhen Xu^2,4*^

^1^Department of Aquatic Animal Medicine, College of Fisheries, Huazhong Agricultural University, Wuhan, Hubei 430070, China

^2^State Key Laboratory of Freshwater Ecology and Biotechnology, Institute of Hydrobiology, Chinese Academy of Sciences, Wuhan, Hubei, 430072, China

^3^College of Fisheries and Life Science, Dalian Ocean University, Dalian 116023, China

^4^Laboratory for Marine Biology and Biotechnology, Qingdao National Laboratory for Marine Science and Technology, Qingdao 266071, China

^†^These authors contributed equally to this work.

^*^Corresponding Author: zhenxu@ihb.ac.cn.


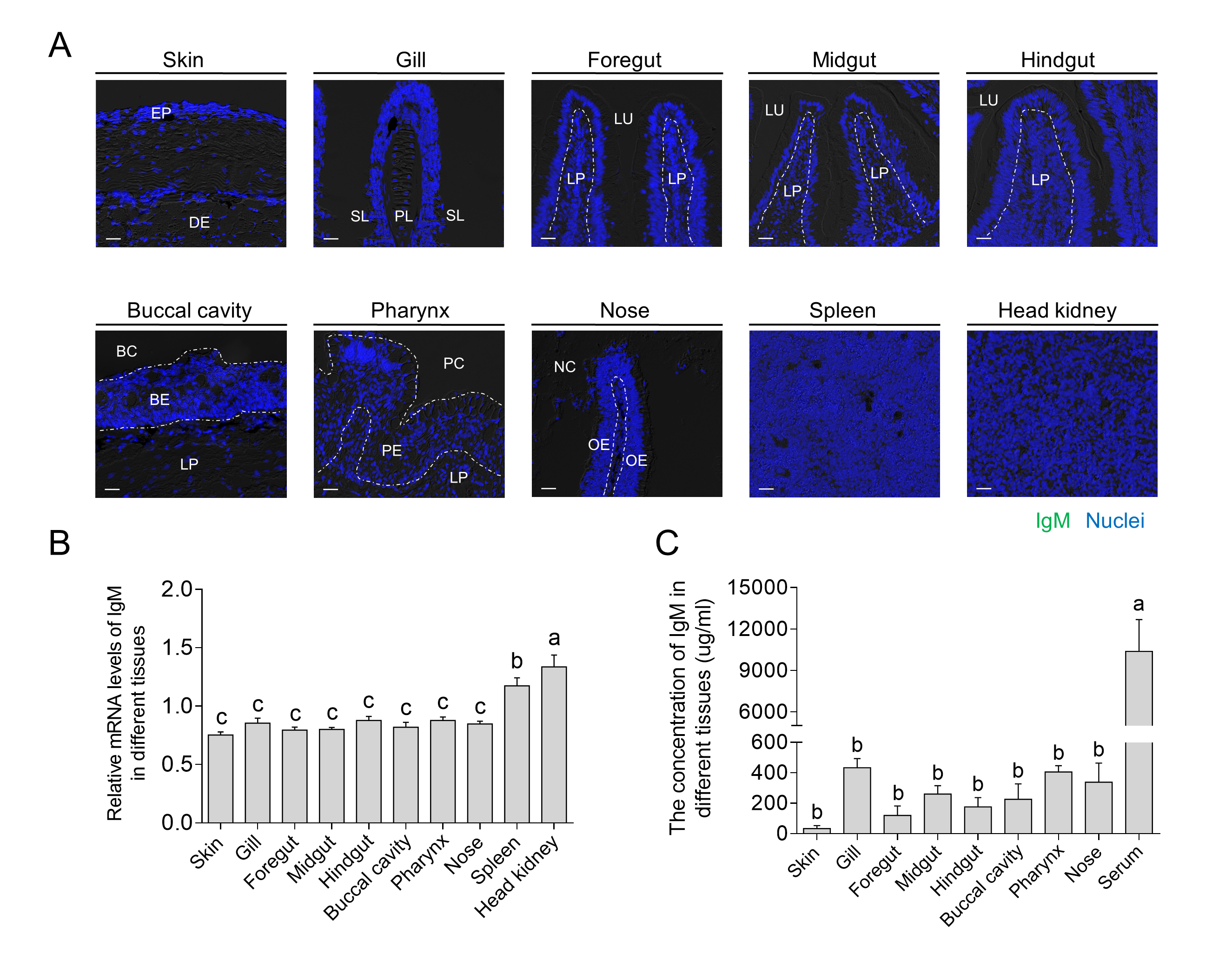


**FIGURE** **S1** | Isotype-matched control antibody staining and IgM distribution in common carp tissues. **(A)** Isotype control staining for anti-IgM (green) antibody in common carp skin, gill, foregut, midgut, hindgut, buccal cavity, pharynx, nose, spleen, and head kidney paraffin sections. Nuclei were stained with DAPI (blue). White dotted lines outline the border. EP, epidermis; DE, dermis; PL, primary lamella; SL, secondary lamella; LU, lumen; LP, lamina propria; BC, buccal cavity; BE, buccal epithelium; PC, pharyngeal cavity; PE, pharyngeal epithelium; NC, nasal cavity; OE, olfactory epithelium. Scale bars, 20 μm. Data are representative of three independent experiments. **(B)** Relative mRNA levels of IgM in the skin, gill, foregut, midgut, hindgut, buccal cavity, pharynx, nose, spleen, and head kidney of healthy common carp were normalized against control gene 40S (*n* = 6). **(C)** The concentration of IgM in mucus and serum from healthy control fish (*n* = 9). Different superscript letters (a, b, c) in each group denote significant variations suggested by the one-way ANOVA with Bonferroni correction (*p* < 0.05). Data are representative of at least three independent experiments (mean ± SEM).


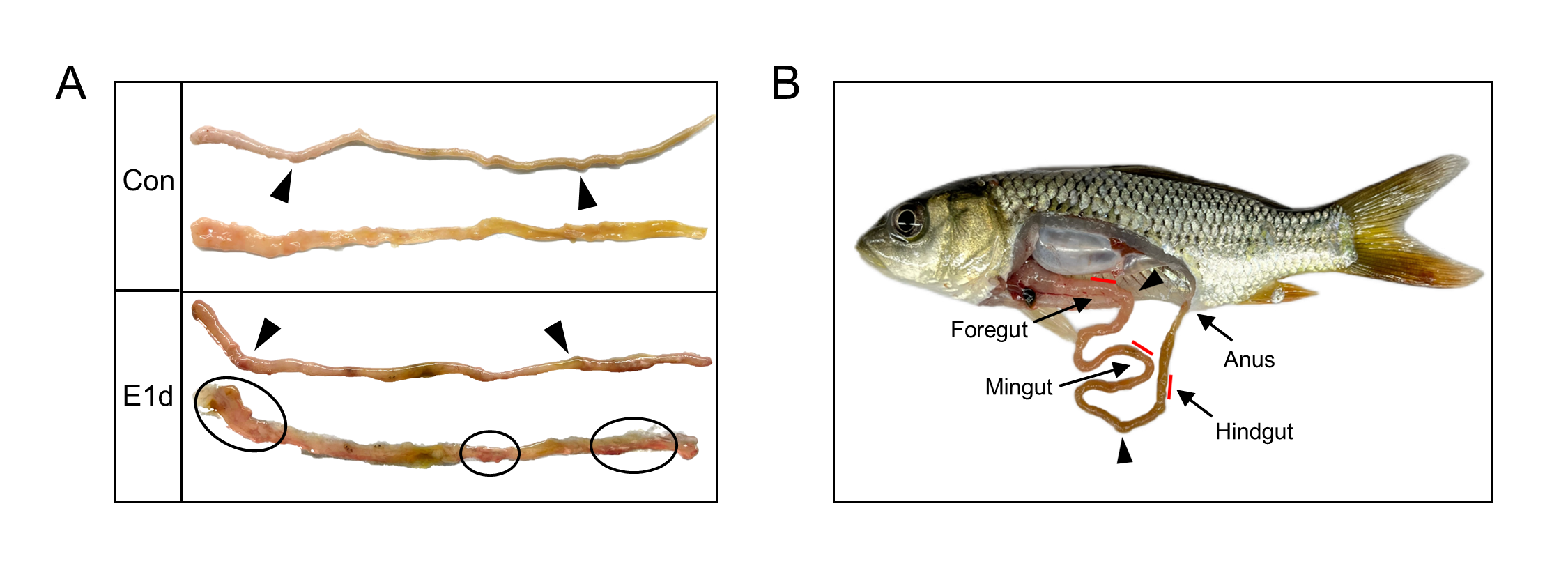


**FIGURE** **S2** | Gut clinical observation and sampling position. **(A)** The gut clinical and opened longitudinally observation at 1 DPI following challenge with *A. hydrophila*. Black circles indicate sites of intestinal inflammation. **(B)** The sampling position of the common carp’s gut. Black triangles in (A and B) indicate the dividing line of the foregut, midgut, and hindgut.


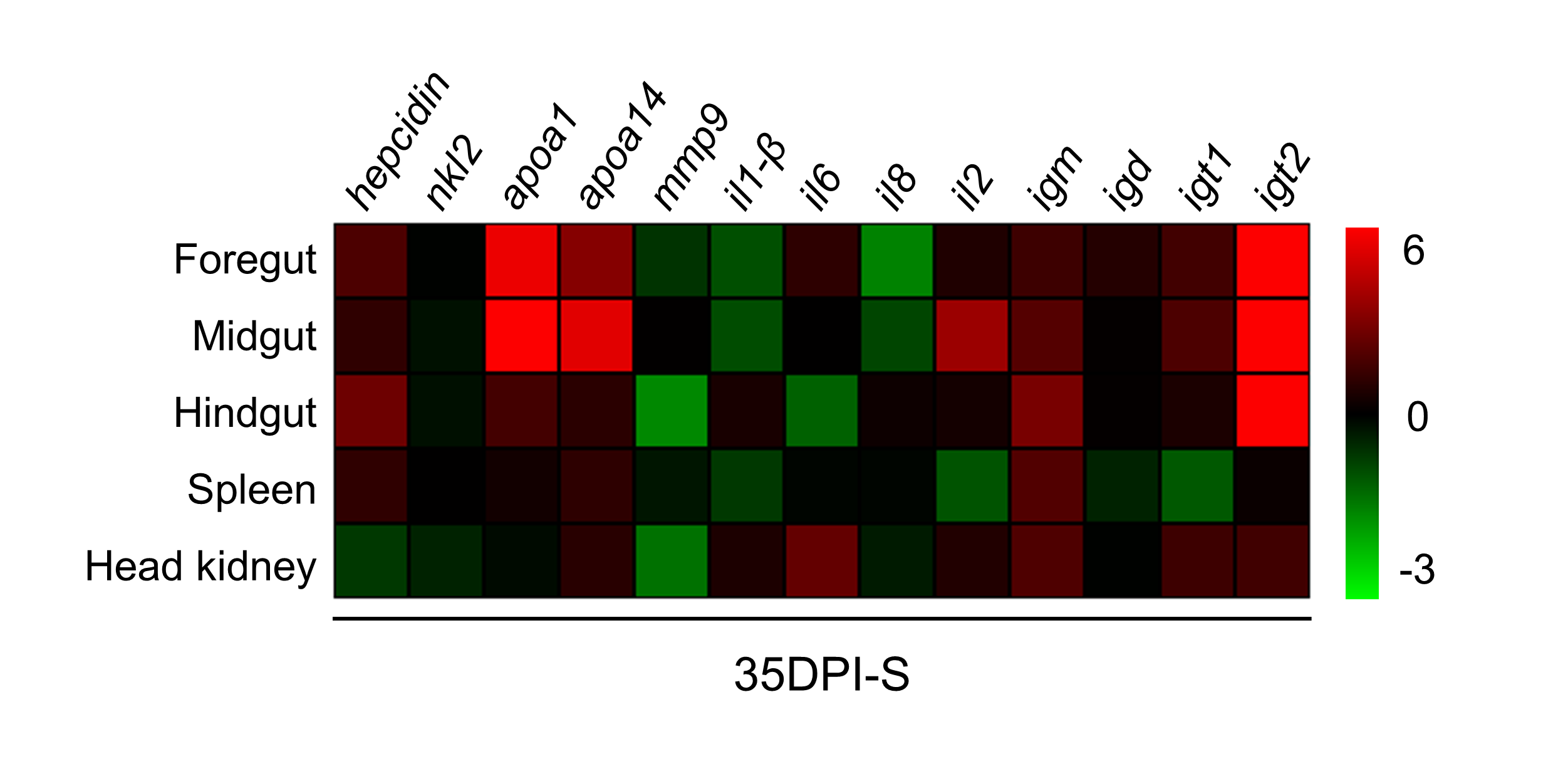


**FIGURE S3** | Immune response in foregut, midgut, hindgut, spleen, and head kidney tissues of common carp post the second infection. Heat map illustrates results from qPCR of mRNAs for selected immune-related genes in 35DPI-S fish vs. control fish (*n* = 6). Color value: log_2_ (fold change). Data are representative of at least three different independent experiments.

**
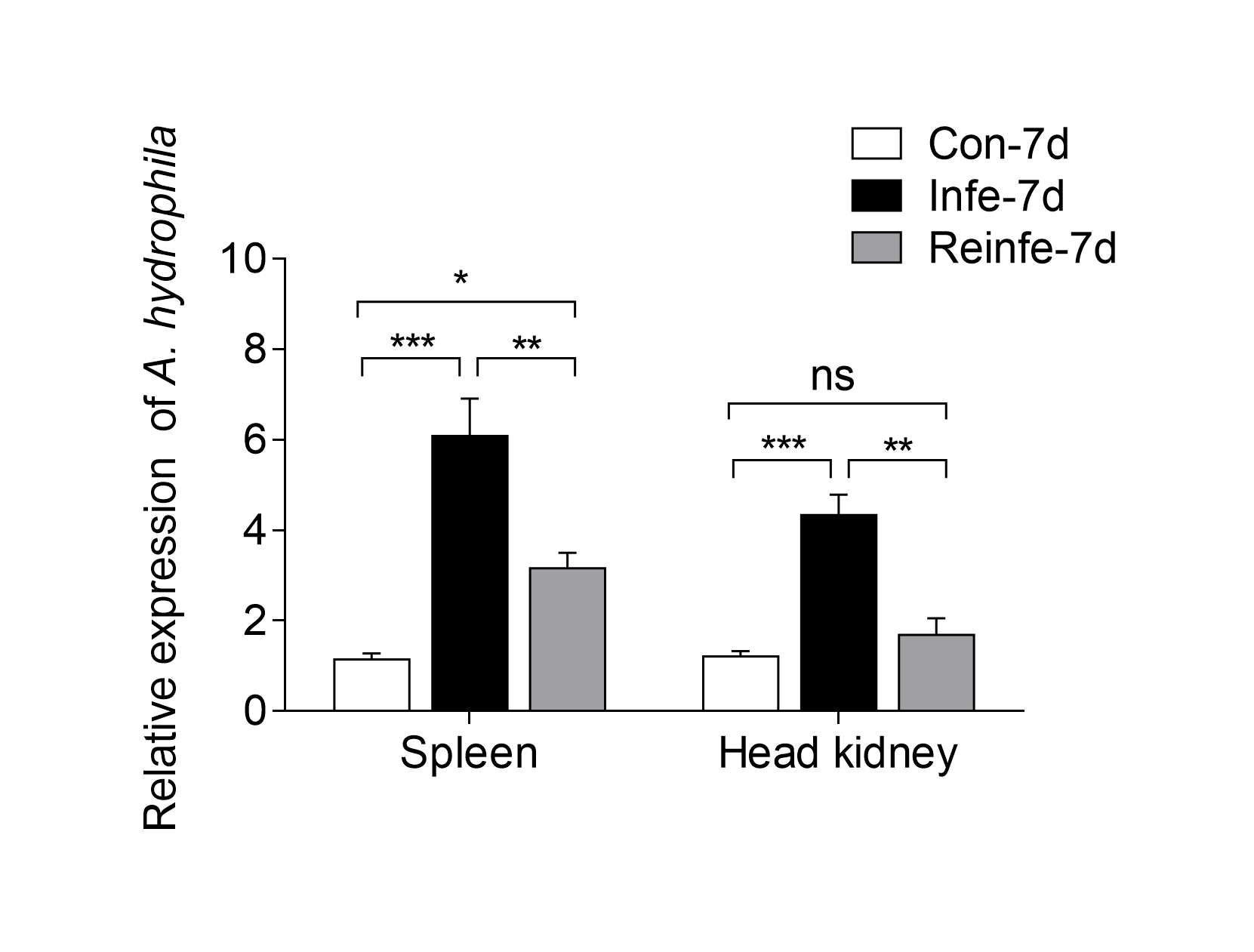
**

**FIGURE** **S4** | The expression levels of *A. hydrophila* in Con-7d, Infect-7d, and Reinfe-7d fish were measured in spleen and head kidney of common carp (*n* = 6). **P* < 0.05, ***P* < 0.01, and ****P* < 0.001 (one-way ANOVA with Bonferroni correction). Data are representative of at least three independent experiments (mean ± SEM).

**TABLE S1** | Gene-specific primers used for qPCR in this study.

| **Gene** | **GenBank**  **accession no.** | **Primer Sequence (5’-3’)** |
| --- | --- | --- |
| *40S* | AB012087.1 | F: CCGTGGGTGACATCGTTACA  R: TCAGGACATTGAACCTCACTGTCT |
| *hepcidin* | KC795559.1 | F: GCATGCGTCTGCATCCTCC  R: CTGGTTCTCCTGTGGTGCTT |
| *nkl2* | KX034213.1 | F: GTCCTGATCACCCTGCTGAT  R: AGCACTTTCCAGGGAGTTGT |
| *apoa1* | AJ308993.1 | F: CCATCTCCGCCTCCTTTC  R: ATGTGTTAGTGTGTGTGTGCTTC |
| *apoa14* | JQ038773.1 | F: CACCAACAGGAGGACAAGCCAAAG  R: GCCATAAGCACCAAGAAGAGCCAAG |
| *mmp9* | AB057407 | F: ATGGGAAAGATGGACTGCTG  R: TCAAACAGGAAGGGGAAGTG |
| *il1-β* | AB010701.1 | F: CAGAGCAACAAACTAAGTGACGAG  R: ACCATCTAACTGGGTACAAGCAAG |
| *il6* | AY102632.1 | F: GTTTACACCCACCTGAAGGAGTT  R: GATTTCTAAGATACAGTTCACCCTCAC |
| *il8* | KU881637.1 | F: GGGTGTAGATCCACGCTGTC  R: AGGGTGCAGTAGGGTCCAGA |
| *il2* | AF486820.1 | F: CGAACGGGACGAGAAATGG  R: TGATAAAGAGCTGCTGTGAATG |
| *igm* | AB004105.1 | F: TAGTGCCTCCCTCCCTTGA  R: AGTGCCGTTGCTCCATTCT |
| *igd* | AB774152.1 | F: TTGGTTGTTGGTCAGAGT  R: TTGGATTGTGAACGATGC |
| *igt1* | AB598367.1 | F: CCAAGAAGGCAACATCATCA  R: AGTGAGGTTCCTGGGGTAGA |
| *igt2* | AB598368.1 | F: CCATGTGCGTATCAGTAAAAGT  R: CTCTAGTGAGGTGCCTTCAGA |
| *A. hydrophila* (Act) | KC687134.1 | F: GTACACGACAGCGCGAATTT  R: ACGTCCATGTCTTCACCGAC |
